# Mutations in *STAG2* cause an X‐linked cohesinopathy associated with undergrowth, developmental delay, and dysmorphia: Expanding the phenotype in males

**DOI:** 10.1002/mgg3.501

**Published:** 2018-11-16

**Authors:** Sureni V. Mullegama, Steven D. Klein, Rebecca H. Signer, UCLA Clinical Genomics Center, Eric Vilain, Julian A. Martinez‐Agosto

**Affiliations:** ^1^ Department of Molecular and Human Genetics Baylor College of Medicine Houston Texas; ^2^ Department of Human Genetics David Geffen School of Medicine University of California, Los Angeles Los Angeles California; ^3^ Division of Medical Genetics, Department of Pediatrics David Geffen School of Medicine University of California, Los Angeles Los Angeles California; ^4^ Department of Pathology and Laboratory Medicine, David Geffen School of Medicine University of California, Los Angeles Los Angeles California; ^5^ Department of Genomic and Precision Medicine Children’s National Hospital Washington District of Columbia; ^6^ UCLA Clinical Genomics Center, David Geffen School of Medicine University of California Los Angeles, Los Angeles California

**Keywords:** clinical exome sequencing, cohesin complex, cohesin‐associated genes, cohesinopathies, human growth, neurodevelopment, reanalysis, *STAG2*, X‐linked gene

## Abstract

**Background:**

The cohesin complex is a multi‐subunit protein complex which regulates sister chromatid cohesion and separation during cellular division. In addition, this evolutionarily conserved protein complex plays an integral role in DNA replication, DNA repair, and the regulation of transcription. The core complex is composed of four subunits: RAD21, SMC1A, SMC3, and STAG1/2. Mutations in these proteins have been implicated in human developmental disorders collectively termed “cohesinopathies.”

**Methods:**

Using clinical exome sequencing, we have previously identified three female cases with heterozygous *STAG2* mutations and overlapping syndromic phenotypes. Subsequently, a familial missense variant was identified in five male family members.

**Results:**

We now present the case of a 4‐year‐old male with developmental delay, failure to thrive, short stature, and polydactyly with a likely pathogenic *STAG2* de novo missense hemizygous variant, c.3027A>T, p.Lys1009Asn. Furthermore, we compare the phenotypes of the four previously reported *STAG2* variants with our case.

**Conclusion:**

We conclude that mutations in *STAG2* cause a novel constellation of sex‐specific cohesinopathy‐related phenotypes and are furthermore, essential for neurodevelopment, human growth, and behavioral development.

## 1 INTRODUCTION

Cohesinopathies are rare multisystem developmental disorders associated with defects in the cohesin complex which enables chromosome segregation and modulates gene transcription (Di Benedetto et al., 2013). The cohesin complex is composed of four cores proteins, SMC1A, SMC2, RAD21, and STAG1/2. The interaction of the cohesin complex with chromosomes is regulated by several proteins (*NIPBL, ESCO2, HDAC8, DDX11, SGOL1*,* WAPL*,* PDS5A, PLK1, AURKB,* and *ATRX*) (Gard et al., 2009). The majority of these genes have been reported in human disease (Ball, Chen, & Yokomori, 2014; Barbero, 2013; Gerkes, Kevie‐Kersemaekers, Yakin, Smeets, & Ravenswaaij‐Arts, 2010; McNairn & Gerton, 2008; Musio & Krantz, 2010; Skibbens et al., 2013). Recently, we described the first reported de novo heterozygous loss‐of‐function variants in the X‐linked Stromal Antigen 2 (*STAG2,* OMIM: 300826) gene, in three females with common phenotypes including microcephaly, microtia, hearing loss, developmental delay, dysmorphic features, language delay, congenital heart defect, and clinodactyly of the 5th finger (Mullegama et al., 2017). Subsequently, a familial *STAG2* germline hemizygous missense mutation in five males was recently identified that exhibited an overlapping yet milder phenotype than our published female *STAG2* cases. This included moderate intellectual deficiency, short stature, peculiar facies, cleft palate, and unilateral deafness (Soardi et al., 2017). Here, we describe the first de novo hemizygous missense *STAG2* variant in a 4‐year‐old male and further define the phenotype of *STAG2* mutations.

## 2 CLINICAL REPORT

**Figure 1:**
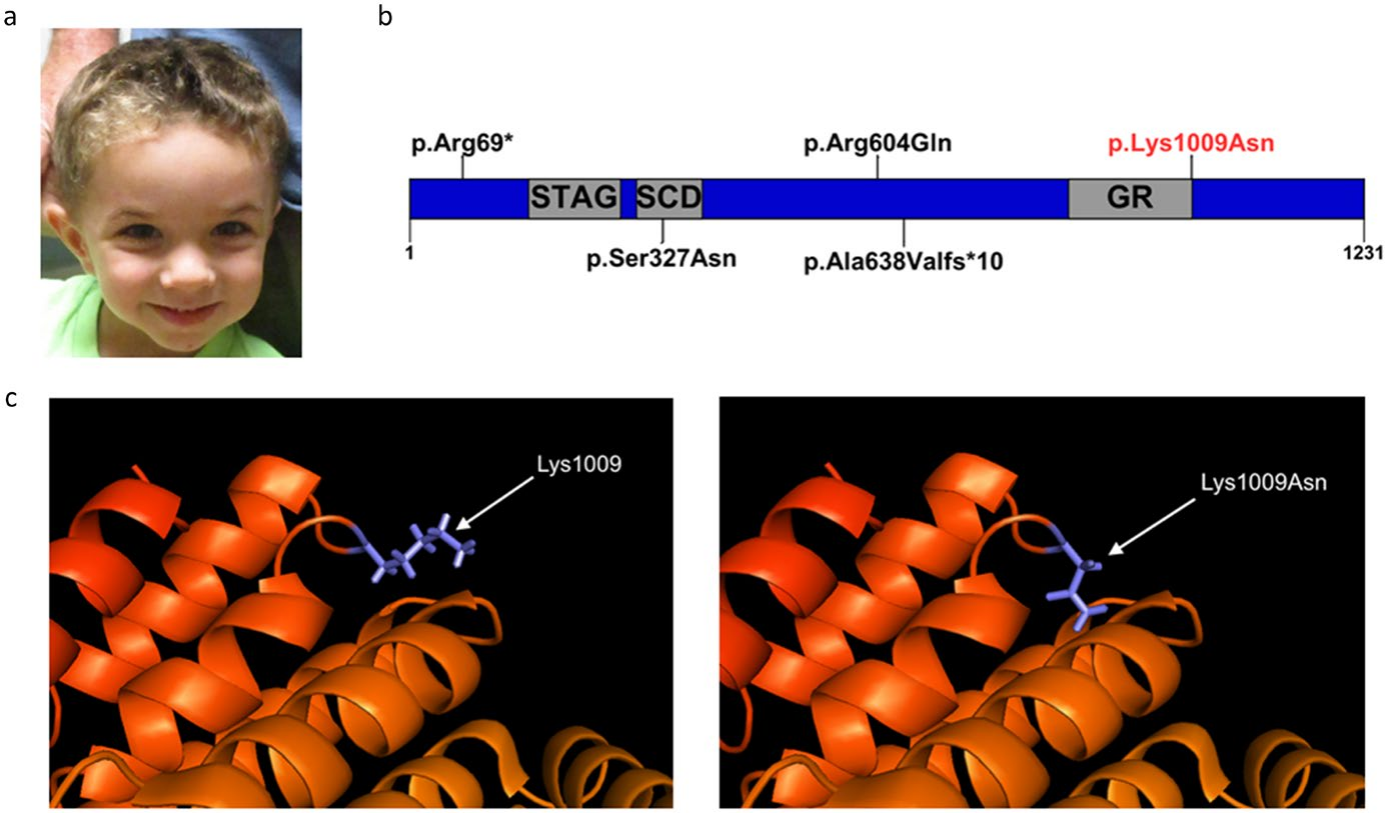
(a) Four‐year‐old male with dysmorphic features consisting of microcephaly, high anterior hairline, mild frontal bossing, prominent cheeks, and triangular face. (b) Schematic representation of the STAG2 protein. The p.Arg69* and p.Ala638Valfs*10 are LOF variants in two females. The p.Ser327Asn is a missense variant present in a female. The p.Lys1009Asn is the proband's variant (in red). (c) Three‐dimensional structural modeling of STAG2 demonstrating effects on protein structure

The proband was born at 39 weeks’ gestation via cesarean section to a 31‐year‐old gravida 3, para 3 mother, and a 37‐year‐old father. Pregnancy was not significant for any major medical problems, teratogenic exposures, or hospitalizations. The parents were of Czech/Slovakia and Scottish/Irish ancestry, and family history was negative for consanguinity. Mother has hypothyroidism, mild hearing loss, attention deficit disorder, and delayed puberty. Depression and anxiety disorder runs on the maternal side of the family. The paternal lineage has a history of schizophrenia. Patient has two other siblings who are healthy, with no reported developmental or physical abnormalities. Birthweight was 1993.71 g (<1st percentile) and length 45.72 cm (<3rd percentile). He presented with early onset failure to thrive. Development was delayed: He crawled at 14 months, independently sat at 13 months, stood up and walked at 16 months, spoke his first words at 13 months, and used small phrases at 17 months. Brain MRI at 1 year of age did not reveal any brain malformations. He had foot surgery when he was 1‐year old to correct polydactyly. He had pes planus requiring orthoses. His audiology testing was normal. At 3 years of age, he started receiving hormone replacement for his growth hormone deficiency. Physical therapy evaluation at this time revealed decreased muscle tone in his lower extremities and abnormalities in gait suggestive of cerebellar dysfunction. At 4‐year‐old weight was 13.9 kg (7th percentile), length was 98 cm (15th percentile), and head circumference was 46 cm (<1st percentile). Dysmorphic features consisted of microcephaly, high anterior hairline, mild frontal bossing, prominent cheeks, triangular face (Figure 1a), and 5th digit clinodactyly. He had speech delay with articulation difficulties and received speech, occupational, and physical therapies. The proband was referred to a genetics clinic to obtain comprehensive cytogenetic and molecular testing. The proband underwent a chromosomal SNP array (Affymetrix Genome‐wide 6.0 SNP‐microarray) which revealed no abnormality. Due to his facial gestalt resembling Russell‐Silver Syndrome, methylation and copy number analysis for chromosome 11p15 and UPD for chromosome 7 testing was performed which were within normal limits. Clinical exome sequencing (CES) did not reveal any known disease‐associated gene variants. Two years after initial CES testing, reanalysis was performed.

## 3 METHODS

### 3.1 Ethical compliance

All samples and information were collected after informed consent was obtained and in accordance with local Institutional Review Board (IRB) approved protocol from the University of California, Los Angeles (UCLA).

### 3.2 Clinical exome sequencing (CES)

Clinical exome sequencing was performed at the UCLA Clinical Genomics Center, on DNA of the proband and both parents, as previously described (Lee et al., 2014).

### 3.3 Pymol modeling of mutation

The STAG two protein file was downloaded from the Protein Data Bank (PBD, File 4PJU), which was then opened in PyMol v1.7.4.5. Protein was visualized in Cartoon Mode, and then, residue 1,009 was selected and cleaned with the “Cartoon Clean Tool.” Residue 1,009 was changed from Lys to Asn using the mutagenesis wizard. The rotomer with the highest likelihood was selected. Associated protein changes were analyzed via selection of all structure within 5 angstroms, and then, the protein was cleaned accordingly (Schrodinger, 2015a, 2015b, 2015c).

## 4 RESULTS

### 4.1 Reanalysis of CES identifies a de novo hemizygous variant in *STAG2*

Within the primary gene list, no established clinically significant single‐nucleotide variants (SNVs) or small deletions and insertions (<10 bp) were identified. Outside of the primary gene list, reanalysis identified a hemizygous c.3027A>T (p.Lys1009Asn) variant in *STAG2* (Genbank: NC_000023.10 (123094475–123236506), Supporting Information Table S1). The variant is absent from public databases including Exome Aggregation Consortium (ExAC) and Genome Aggregation Database (gnomAD). The ExAC population database predicts that *STAG2* is depleted of missense variants in healthy populations (90 observed missense variants compared to the 258 expected missense variants), suggesting that this class of variants may be deleterious. Functional prediction algorithms such as Poly‐Phen and Sift predict this variant to be damaging. The evolutionarily conserved amino acid position is located at the end of the evolutionarily conserved GR functional domain (Figure 1b,c) (Schrodinger, 2015a, 2015b, 2015c ). The variant was initially classified as a variant of unknown clinical significance based on the American College of Medical Genetics and Genomics variant assessment (Richards et al., 2015) (Supporting Information Table S1). Since then, *STAG2* variants have been recently been associated with a new clinical entity (Mullegama et al., 2017; Soardi et al., 2017). Based on the effects of the missense variant on the protein structure (Figure 1c), this variant, p.Lys1009Asn, was reclassified as likely pathogenic. Analysis of the reads covering the same base from CES in the mother and father confirms no evidence of even low abundance mutation, confirming that the mutation is de novo in the proband (Supporting Information Figure S1a–c).

## 5 DISCUSSION

This case further confirms that pathogenic variants in *STAG2* result in a novel cohesinopathy. The first reports of *STAG2* copy number variants were identified in 33 males with chromosome Xq25 duplications, involving *STAG2,* with intellectual disability, behavioral problems, seizures, malar flatness, and prognathism (Bonnet et al., 2009; Kumar et al., 2015; Leroy et al., 2016; Philippe et al., 2013; Yingjun et al., 2015). Shortly after, we described three cases of de novo heterozygous *STAG2* variants in females associated with a distinct phenotype that affects growth, nervous system development, hearing, and limb formation (Table 1) (Mullegama et al., 2017). Subsequently, a familial *STAG2* missense mutation was identified in one family with five affected males. These five cases presented with moderate cognitive delay, growth retardation, peculiar facies, cleft palate, and unilateral deafness (Soardi et al., 2017). To further solidify the gender‐specific phenotype associated with *STAG2* hemizygosity, we compared the phenotype of our proband to female *STAG2* LOF pathogenic variants and also to the established cohesinopathies (Table 1 and Supporting Information Table S2). We demonstrate that they share the core clinical findings of cognitive delay, growth retardation, neuropsychiatric behaviors, microcephaly, craniofacial dysmorphia, and syndactyly (Supporting Information Table S2). It appears that decreased dosage of *STAG2* has a more severe phenotype than increased dosage of *STAG2* (Table 1)*.* However, the discovery of additional patients with *STAG2* pathogenic variants will help identify whether there is a dosage effect of *STAG2* on phenotype. Further, we theorize that decreased dosage of *STAG2* is associated with a neurodevelopmental phenotype, growth retardation, and craniofacial dysmorphia.

**Table 1:** Comparison of *STAG2* variants

	De novo missense	Familial missense[Link]	De novo LOF[Link]	CNVs[Link]
Mutation	p.Lys1009Asn	p.Ser327Asn	p.Arg69[Link]	Duplications
Gender	Male	Male	Female	Males
Phenotypes				
Cognitive delay	+ (mild–moderate)	+ (moderate)	+	+ (mild–moderate)
Failure to thrive	+	NA	+	−
Growth retardation	+	+	+	−
Language delay	+	NA	+	−
Neuropsychiatric behaviors	+	NA	+	+
Microcephaly	+	NA	+	−
Craniofacial dysmorphia	+	+	+	+
Cleft/arched palate	−	+	+	−
Digit abnormalities[Link]	+	−	+	−
MRI abnormalities	−	NA	+	−
Scoliosis	−	−	+	−
Cardiac defects	−	−	+	−
Hearing loss	−	+ (unilateral)	+	−

Notes

NA: no clinical information was provided.

Soardi et al. (2017).

Mullegama et al. (2017).

Bonnet et al. (2009), Di Benedetto et al. (2013), Leroy et al. (2016), Philippe et al. (2013) and Yingjun et al. (2015).

Polydactyly, syndactyly and 5th finger clinodactyly.

The utilization of exome sequencing has led to the discovery of novel X‐linked genetic disorders (Retterer et al., 2016; Yang et al., 2013). The clinical severity of females and males is most likely dependent on (a) the type of variant (missense vs. truncation); (b) location (within a functional domain); or (c) skewed X‐chromosome inactivation (XCi) (Chae, Hwang, Hwang, Cheong, & Kim, 2004; Weaving et al., 2003). We had previously postulated that complete loss‐of‐function hemizygous variants that affect canonical STAG, SCD, or GR domains would not be identified in males because *STAG2* is an X‐linked gene and complete loss would result in embryonic lethality (Mullegama et al., 2017). The pLys1009Asn variant found in our male proband is located on the protein surface (Figure 1). It is possible that this variant affects protein stability, or interactions and ultimate assembly into the cohesion complex. We suspect that this mutation, based on surface location, may decrease the affinity of protein–protein or protein–DNA interactions, however, does not ablate them, which might explain the milder phenotype. In addition, the variant reported by Soardi et al. (2017) which was observed in male probands and shown to decrease the interaction between STAG2 and SCC1 which supports this hypothesis (Soardi et al., 2017).

Upon comparing the limited *STAG2* patient cohort, we propose the following model. Females, who carry two copies of STAG2, are able to survive deleterious de novo variants, however display severe phenotypes. Males are unable to survive similar variants due to early embryologic lethality. Males are able to survive less damaging variants and as a result present with milder phenotypes. On the spectrum of male STAG2‐related cohesinopathies, we also see milder phenotypes in patients with chromosome Xq25 duplications suggesting that dosage and function are essential for proper cohesion complex function. Similar dosage effects have been established for *MECP2* (OMIM: 300005).

We believe that cohesinopathy/gene‐specific panels may be utilized by clinical geneticists when evaluating male patients with phenotypic constellations similar to the case herein reported. The major criteria are shared by all cohesinopathies and include cognitive delay, growth retardation, neuropsychiatric behavior abnormalities, microcephaly, dysmorphia, and digit anomalies. The true phenotypic spectrum will be expanded upon the discovery and reporting of additional male cases.

In conclusion, we report the first male de novo variant in *STAG2* and confirm that its function is essential for human growth and behavioral development. Overall, STAG2 is essential for human growth and behavioral development. The future identification of additional pathogenic variants in *STAG2* will allow us to further understand the phenotypic spectrum of this novel cohesinopathy.

## CONFLICT OF INTEREST

No conflict of interests.
