# Supplementary figures and images for: Mutations in STAG2 cause an X‐linked cohesinopathy associated with undergrowth, developmental delay, and dysmorphia: Expanding the phenotype in males
Source: Mol Genet Genomic Med. 2018 Nov 16;7(2):e00501. doi: 10.1002/mgg3.501 (PMC6393687; doi:10.1002/mgg3.501)

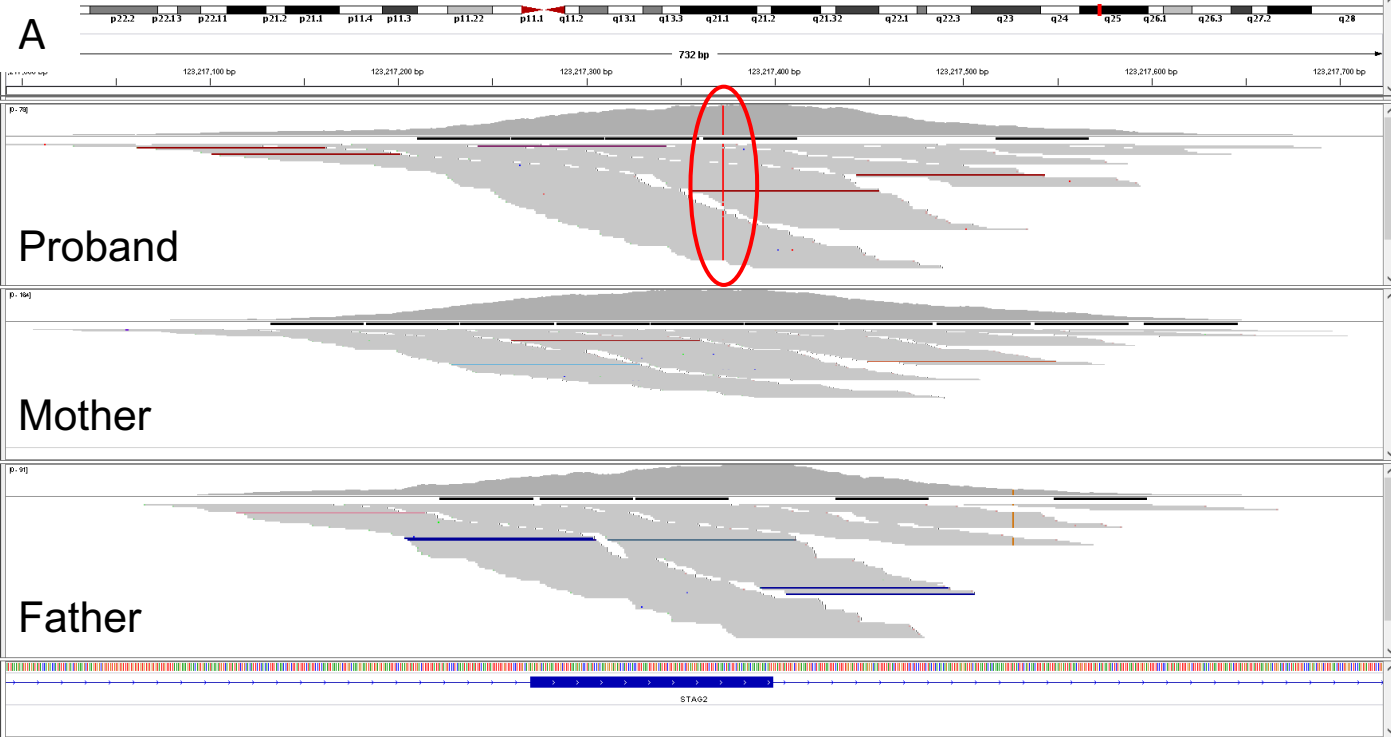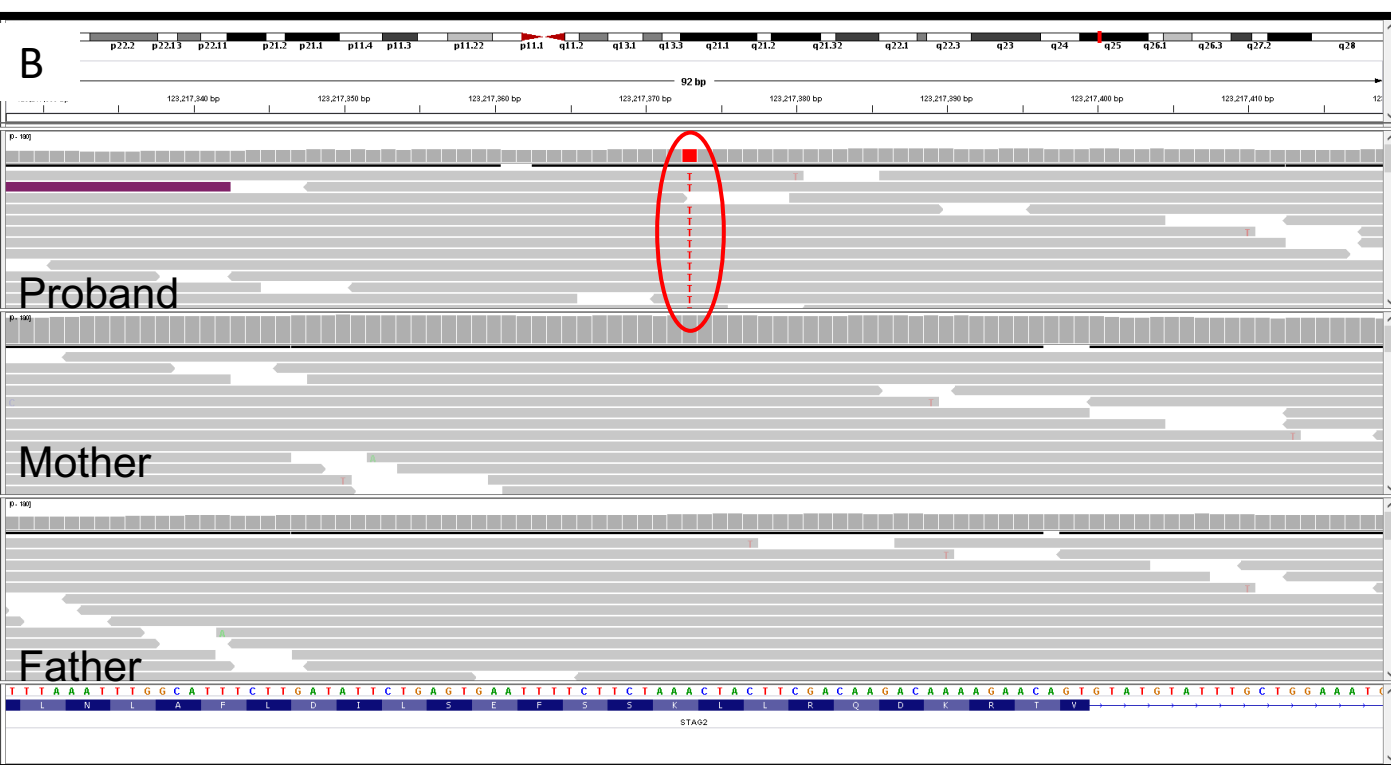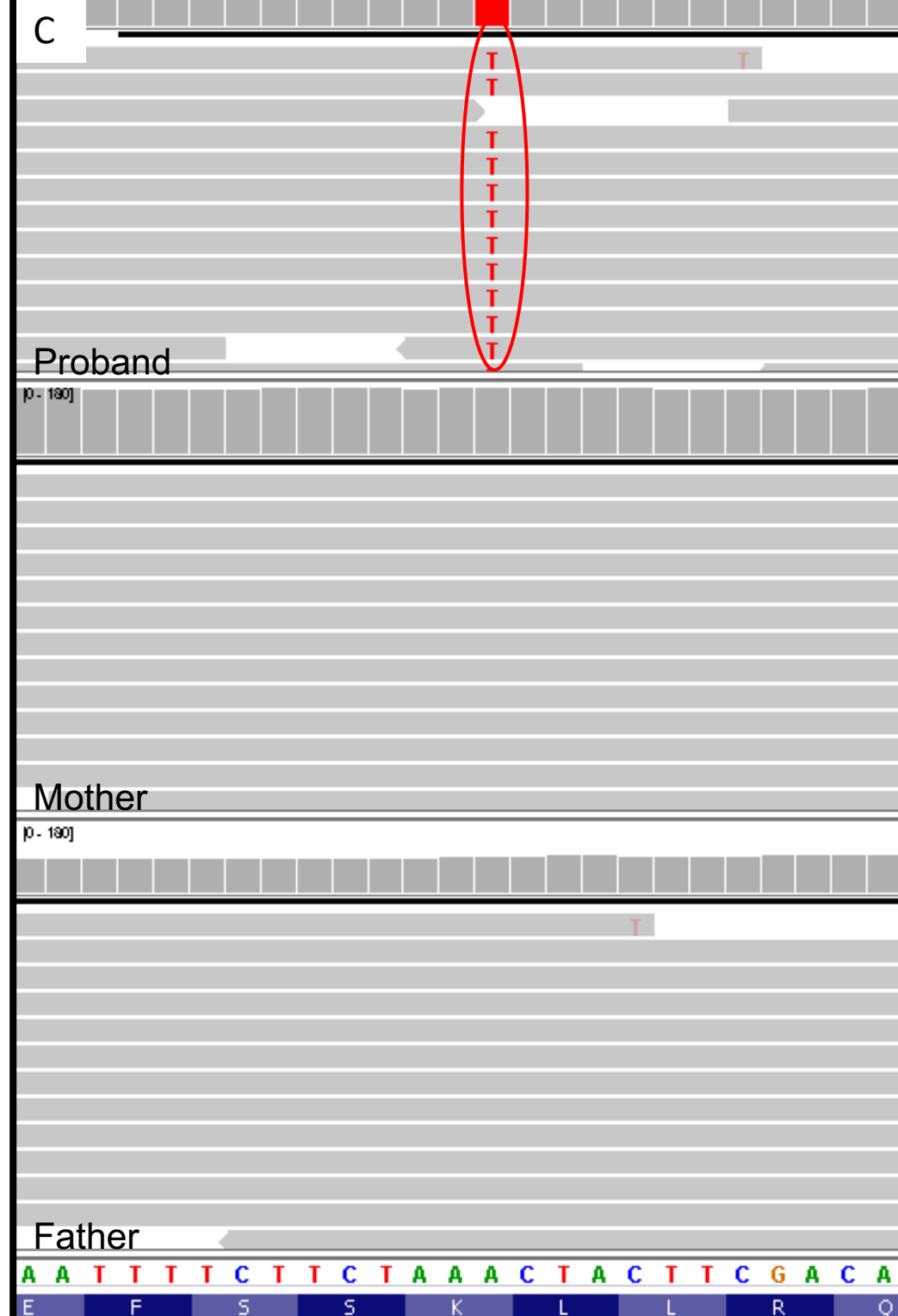

Supplement: Supplementary file 1 [file MGG3-7-na-s001.pdf]
