# Supplementary material for: Mutations in STAG2 cause an X‐linked cohesinopathy associated with undergrowth, developmental delay, and dysmorphia: Expanding the phenotype in males
Source: Mol Genet Genomic Med. 2018 Nov 16;7(2):e00501. doi: 10.1002/mgg3.501 (PMC6393687; doi:10.1002/mgg3.501)
Supplement: Supplementary file 2 [file MGG3-7-na-s002.docx]

**Mutations in *STAG2* Cause an X-linked Cohesinopathy Associated With Undergrowth, Developmental Delay And Dysmorphia: Expanding the Phenotype in Males**

Sureni V. Mullegama^1,2#^*,* Steven D. Klein^3#^, Rebecca Signer^4^, UCLA Clinical Genomics Center^2^, Eric Vilain^2,3,4^ and Julian A. Martinez-Agosto^2,3,4*^

^1^Department of Pathology and Laboratory Medicine, David Geffen School of Medicine, University of California, Los Angeles, Los Angeles, California, USA

^2^UCLA Clinical Genomics Center, David Geffen School of Medicine, University of California, Los Angeles, Los Angeles, California, USA

^3^Department of Human Genetics, David Geffen School of Medicine, University of California, Los Angeles, Los Angeles, California, USA

^4^Department of Pediatrics, David Geffen School of Medicine, University of California, Los Angeles, Los Angeles, California, USA

#These authors contributed equally to this work

**Conflict of interest:** No conflicts of interests

**^*^Correspondence to:**

Julian A. Martinez-Agosto MD, PhD

Address: 695 Charles E. Young Drive South, Gonda Research Center Room 4605, Los Angeles, CA 90095

Phone: 310-794-2405

Fax: 310-794-5446

Email: julianmartinez@mednet.ucla.edu

**Supplementary Table 1.** *STAG2* variant identified through clinical whole exome sequencing

| **Variant** | | | **Position** | | | **Variant Type** | | | | **Allele frequency*** | | | | | | **Putative impact** | | **Conservation** | **Experimental evidence** | **Loss of function^+^** | **ACMG guidelines** |
| --- | --- | --- | --- | --- | --- | --- | --- | --- | --- | --- | --- | --- | --- | --- | --- | --- | --- | --- | --- | --- | --- |
| Genomic  Change (hg19) | cDNA change | Protein  change | | Location | Protein domain | | *De novo* variant | Zygosity | Novel | | ExAC | gnomAD | 1000 genomes | | Polyphen2 | | SIFT | Conservation | Experimental evidence |  |  |
| ChrX:g123217373A>T^1^ | c.3027A>T | p.Lys10009Asn | | Exon27 | GR | | Yes | Hemizygous | Yes | | 0 | 0 | 0 | Damaging | | | Damaging | Yes | No | Yes | Likely Pathogenic |
| *Minor allele frequencies listed are from ExAC browser (exac.broadinstitute.org), gnomAD browser beta (gnomad.broadinstitute.org), and 1000 Genomes Project ([www.1000genomes.org](http://www.1000genomes.org))  ^1^ Genbank reference sequence: NC_000023.10 | | | | | | | | | | | | | | | | | | | | | |

| **Supplementary Table 2. Phenotype Comparison of *STAG2* Variants to other Cohesinopathies** | | | | | | | | |
| --- | --- | --- | --- | --- | --- | --- | --- | --- |
|  | **Male Missense variant** | **Female LOF variant** | **CdLS** | **RBS** | **WBS** | **NBS** | **FA** | **CdLS 4** |
| **Gene** | *STAG2* | *STAG2* | *NIPBL*  *SMC1A*  *HDAC8*  *SMC3* | *ESCO2* | *DDX11* | *NBN* | *FANCA*  *&*  others | *RAD21* |
| **Phenotypes** |  |  |  |  |  |  |  |  |
| Cognitive delay | + | + | + | + | + | + | + | + (mild) |
| Growth retardation | + | + | + | + | + | + | + | + |
| Neuropsychiatric behaviors | + | + | + | + | + | + | + | + |
| Microcephaly | + | + | + | + | + | + | + | + |
| Craniofacial dysmorphia | + | + | + | + | + | + | + | + |
| Cleft/arched palate | - | + | + | + | + | + | - | + |
| Syndactyly | + | + | + | + | + | + | - |  |
| Organ abnormalities | - | + | + | + | + | + | + | + |
| Cardiac defects | - | + | + | + | + | - | + | - |
| Limb reductions | - | - | + | + | - | + | + | - |
| Hearing loss | - | + | + | - | + | - | + | - |
| Skin pigmentation abnormalities | - | - | + | - | + | + | + | - |
| Elevated Cancer incidence | - | - | - | - | - | + | + | - |
| Bone marrow/hematopoietic defects | - | - | - | - | - | - | + | - |
